# Long‐term outcome of epileptic dogs treated with implantable vagus nerve stimulators

**DOI:** 10.1111/jvim.16908

**Published:** 2023-10-20

**Authors:** Thomas R. Harcourt‐Brown, Michael Carter

**Affiliations:** ^1^ Langford Vets University of Bristol School of Veterinary Sciences Bristol UK; ^2^ Bristol Royal Hospital for Children University Hospitals Bristol and Weston NHS Foundation Trust Bristol UK

**Keywords:** dog, epilepsy, seizures, vagus nerve

## Abstract

**Background:**

The long‐term effect of implantable vagus nerve stimulators (VNS) on seizures has not been evaluated in epileptic dogs.

**Objectives:**

Report seizure frequency in medication‐resistant epileptic dogs before and after VNS implantation.

**Animals:**

Twelve client‐owned dogs with idiopathic epilepsy and >1 seizure day per 3 weeks despite 3 months of appropriate use of 2 antiseizure medications and seizure diaries maintained 6 months before and >12 months after VNS implantation.

**Methods:**

Uncontrolled, open‐label, before and after study. Mean monthly seizures and inter‐seizure periods obtained from contemporaneous seizure diaries in the 6 months before implantation were compared with 0 to 6 months, 7 to 12 months, and subsequent 12‐month periods after implantation. The number of dogs with >50% decrease in seizure frequency, >3 times increase in inter‐ictal period interval, and seizure freedom for >3 months at the time of death or last follow‐up were recorded.

**Results:**

Five of 12 dogs were euthanized <12 months after implantation. All 7 remaining dogs showed >50% decrease in seizure frequency until last follow‐up, starting at a median of 37 to 48 months after implantation (range, 0‐6 to 61‐72 months) and a >3‐fold increase in mean inter‐seizure interval starting a median of 25 to 36 months after implantation (range, 0‐6 months to 49‐60 months), 3/7 dogs were seizure‐free at death or last follow‐up.

**Conclusions and Clinical Importance:**

Monthly seizure frequencies decreased and inter‐seizure intervals increased in all dogs 2 to 3 years after VNS implantation, but a high proportion were euthanized before this time point. Prospective clinical trials are required to establish causality and the magnitude of this association.

**Abbreviations**
MRI: magnetic resonance imaging
VNS: vagus nerve stimulator or stimulators

## 1 INTRODUCTION

Epilepsy is a common neurological problem in dogs. It is estimated to affect 0.57% to 0.67% of dogs in the United Kingdom, with approximately 88% of these dogs receiving antiseizure medication.^1^ Dogs that do not respond to medication are termed medication‐resistant epileptics. A precise definition for medication resistance is not universally established, but it has been suggested that failure to achieve seizure freedom despite adequate therapeutic trials of 2 appropriate medications would be consistent with medication resistance.^2^ Failure to achieve >50% decrease in seizure frequency is another commonly used definition.^3^

No longitudinal studies of epileptic dogs are available to provide a reliable estimate of medication resistance by either definition, but pooled data for dogs treated with what are thought to be the most effective antiseizure medications (phenobarbital or primidone plus potassium bromide) indicate that failure to achieve seizure freedom occurred in 78% (54/69) and a >50% frequency decrease was not seen in 31% (58/186).^3^

The number of pet dogs in the United Kingdom also is not well established, with estimates ranging from 4.8 to 16.4 million.^4^ Such a population means there could be as many as 110 000 epileptic dogs, with 86 000 of them continuing to seizure despite appropriate medications and 34 000 not having a >50 decrease in seizure frequency. These high numbers clearly indicate a need for alternative treatments in medication‐resistant epileptic dogs.

Implantable vagus nerve stimulators (VNS) have been used as a non‐pharmacological treatment for medication resistant human epileptics for >30 years.^5^ The method of action is not definitively established but is thought to result from decreased seizure generation within affected areas of the brain, either by alteration of the neurotransmitter environment or an anti‐inflammatory process.^6^

Vagus nerve stimulators implanted in epileptic dogs as part of a blinded, controlled, prospective clinical trial were not found to have a significant effect on seizure frequency.^7^ This outcome may be explained by the low stimulation current used in the blinded portion of the study and short (13‐week) assessment period. Our aim was to assess the effectiveness of VNS for seizure reduction in dogs with idiopathic epilepsy when measured over a longer period (>12 months) and used at a higher level of stimulation.

## 2 MATERIALS AND METHODS

For this uncontrolled, open‐label, before and after study, dogs were prospectively recruited from cases referred to Langford Veterinary Services, Bristol, UK if they had a diagnosis of idiopathic epilepsy consistent with a tier‐2 confidence level of diagnosis as defined by the International Veterinary Epilepsy Task Force.^8^ This classification entailed a history of repeated generalized seizures, normal inter‐ictal examination, normal blood analysis (including CBC, serum sodium, potassium, chloride, calcium, phosphate, alanine aminotransferase, alkaline phosphatase, total bilirubin, urea, creatinine, total protein, albumin, glucose, cholesterol, triglycerides, and 2‐hour post‐prandial serum bile acid measurements), unremarkable brain magnetic resonance imaging and normal cisternal cerebrospinal fluid analysis. Dogs were included if they had an average seizure frequency of >1 seizure day per 3 weeks despite the appropriate use of at least 2 antiseizure medications for the 3 months before implantation and were considered medication‐resistant. These 2 medications were required to be phenobarbital and potassium bromide unless the adverse effects of either had been intolerable. Appropriate use was defined as maintaining serum concentrations of the medications within the therapeutic range given by the reference laboratory used for monitoring, and dosing of phenobarbital changed from every 12 hours to every 8 hours if the elimination half‐life was <20 hours.^9^ If adverse effects were found to be intolerable, imepitoin (>20 mg/kg q12h), zonisamide (>5 mg/kg q12h), or levetiracetam (>15 mg/kg q8h) were considered appropriate alternatives. Medication alteration was permitted, and discontinuation or addition of new agents was recorded.

All dogs had VNS placed under general anesthesia. Coil electrodes (model 302 or 303 lead, LivaNova Inc, Houston, Texas) were placed around the cervical portion of the left vagus nerve as described previously.^10^ Generator (Pulse 102 or Aspire HC 105, LivaNova Inc, Houston, Texas) placement either was SC over the dorsal cervical region^10^ or over the lateral cranial thorax just caudal to the position of the scapula. In cases with thoracic placement, the leads extended caudally across the scapula from the electrode implantation side.

Initial settings were 25 Hz pulses of 0.25 mA current with 250 μs pulse width interval delivered for either 30 seconds every 5 minutes in 3 dogs or 7 seconds every 1.8 minutes in the remaining dogs to decrease the severity of stimulation‐induced coughing.^11^ Post‐operatively, the stimulator current was increased to 1.5 mA in 0.25 mA steps every 8 to 12 hours (fast‐ramping) or every 1 to 3 weeks (slow‐ramping) This current was chosen to be consistent with recommendations made for humans because it is thought to most effectively depolarize axons in the vagus nerve.^12^

If severe coughing or any other intolerable stimulation‐related adverse effects were encountered during ramping, signal frequency was decreased (to a minimum of 20 Hz), the on/off cycle was changed from 30 seconds every 5 minutes to 7 seconds every 1.8 minutes (if dogs were not already on this regimen) or the current decreased to the previously tolerated level, and attempts made to increase at a later time. This protocol is discussed in more detail elsewhere.^11^

Dogs were followed up with face‐to‐face consultations as current was increased. Once a current of 1.5 mA was reached, further face‐to‐face consultations were performed when adverse effects of the VNS were suspected, when high seizure frequencies prompted attempts to increase stimulator current or decreased off time, when VNS failure was suspected because of abrupt cessation in coughing, or at least every 12 to 18 months.

Dates of seizures and inter‐seizure intervals were collected from owner‐compiled seizure diaries for 12 months pre‐implantation and at least 12 months post‐implantation. The type of seizure diary used (eg, handwritten or electronic) was at the owners' discretion. Data from diaries was collected at reprogramming appointments, or other telephone, email or face‐to‐face consultations at least every 18 months.

For the purposes of all analyses, 1 month was considered contain 30 days (365/12) and 1 year to contain 365 days. Only generalized seizures were counted. Seizure days were considered to be any calendar day containing ≥1 reported seizure. If >1 seizure occurred during a calendar day or at least 1 seizure occurred on consecutive days, it was considered a cluster seizure.

Mean monthly seizure days for each dog were calculated for a 6 to 12 month and 0‐ to 6‐month period pre‐implantation and 0 to 6 months, 7 to 12 months, and subsequent 12‐monthly periods until death or last follow‐up post‐implantation. Mean monthly seizure days were not calculated for time periods where data was missing for >50% of that period (eg, if a dog was euthanized or data were missing from a seizure diary). For each time period post‐implantation, dogs were compared with the 6‐monthly pre‐implantation baseline and categorized as worsened (if mean monthly seizure days increased), improved (if mean monthly seizure days decreased), and responders (if mean monthly seizure days decreased ≥50% compared with baseline), or seizure‐free if no seizure days occurred during this period until death of the end of follow‐up.

Non‐statistical post hoc comparison of seizure day frequencies was made between dogs euthanized before 12 months post‐implantation and those surviving longer to generate hypotheses about why this outcome might have occurred. The proportions with improved mean seizure day frequency in the 6 months post‐implantation were calculated, as well as the median seizure day frequency pre‐ and post‐implantation where data were available. This information was not analyzed statistically because analysis was post hoc and the data sets too small to be reliable.

Mean inter‐ictal interval was defined as the number of days between a solitary seizure day or the last seizure day in a cluster and the next seizure day. It also was calculated in the 6 to 12 month and 0 to 6 month periods pre‐implantation and 0 to 6 months, 7 to 12 months, and then yearly post‐implantation if there was data for >50% of that time period. For each time period post‐implantation, dogs were compared with the 0‐ to 6‐month pre‐implantation baseline and categorized as decreased (if mean inter‐ictal interval decreased), increased (if mean inter‐ictal interval increased) or increased by >3 times baseline (if mean inter‐seizure interval increased >3 times).This last definition was considered relevant because previous mathematical modeling suggested that a seizure‐free period >3 times the maximum previously observed seizure‐free period indicated a 95% certainty that seizure frequency has decreased by ≥50%.^13^

## 3 RESULTS

Vagus nerve stimulators were placed in 16 dogs that fulfilled inclusion criteria from February 2017 to October 2020. The owners of 4 dogs could not maintain a seizure diary. One of the remaining dogs had unrecorded seizure data from 29 to 34 months post‐implantation but was included in analysis, The remaining 11 dogs had complete seizure records (ie, 12 dogs were included for analysis).

Five of the remaining 12 dogs were euthanized at their owners' request for seizure‐related causes. The reasons were perceived lack of response (3 dogs) or a severe cluster of seizures after a period of decreased seizures (2 dogs). The median time to euthanasia for these dogs was 256 days (range, 155‐307). All but 1 of these 5 had received a current ≥1.5 mA.

**FIGURE 1:**
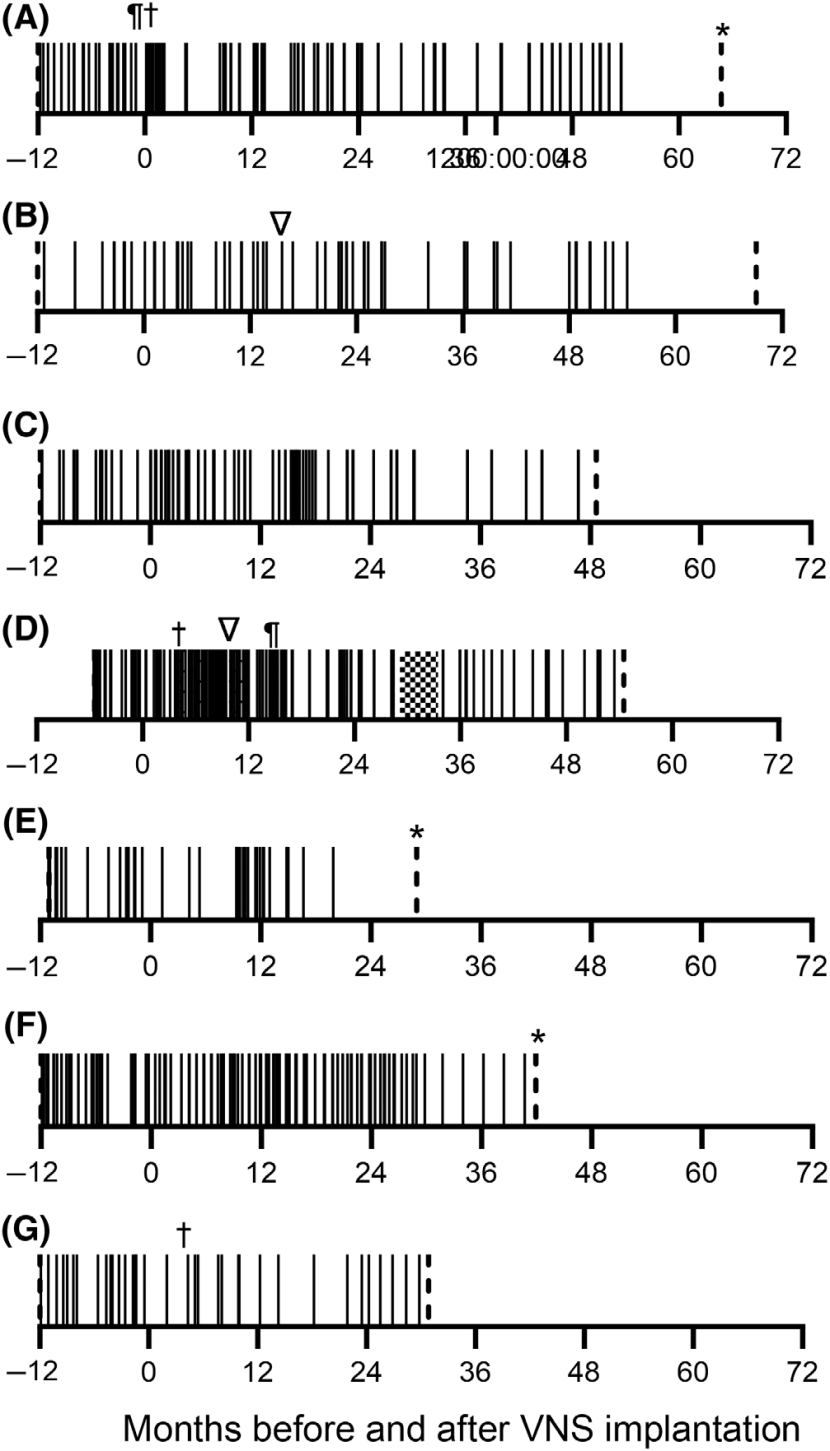
Seizure days recorded for each dog surviving >12 months post‐implantation. Each solid vertical line represents a day with ≥1generalised seizures. A vertical dashed line represents the first or last entry in the seizure diary. ¶ indicates cessation of 1 antiseizure drug, † indicates introduction of a new antiseizure drug, ∇ indicates replacement of the VNS generator or lead or both.

Outcome beyond 12 months was available in 7 dogs (Figure 1). Two dogs were euthanized for causes unrelated to their seizures: 1 for arthritis 29 months post‐implantation and 1 for non‐ambulatory paraparesis 66 months post‐implantation. The remaining 5 dogs were alive at the time of writing with a median follow‐up of 49 months (range, 29‐70).

All dogs were treated with phenobarbital at the time of implantation and at least 1 other appropriate antiseizure medication (Data S1). Three of the 12 dogs had medications changed post‐implantation. One dog (Figure 1A) developed ataxia and obtundation secondary to bromism, bromide was discontinued and gabapentin and zonisamide were begun on day 27 post‐implantation; another dog (Figure 1D) was started on imepitoin on day 121 post‐implantation and levetiracetam was stopped on day 450 because of perceived inefficacy; the third dog (Figure 1G) was started on pregabalin on day 83 to accompany dose reductions in phenobarbital and bromide because of ataxia.

Transient surgery site seromas were seen in 8/12 dogs and dysphagia and tongue paresis were observed in 1 dog (Figure 1D) and occurred during fast ramping but resolved with current reduction and slower increase to 1.5 mA. This outcome is described in more detail elsewhere.^11^

Coughing was the only other adverse event seen. One dog was euthanized at a current of 0.75 mA with mild coughing. After ramping to 1.5 mA by 3 months post‐implantation, 4 dogs never coughed, 4 showed moderate coughing, and 3 showed mild coughing. Two of the 4 with moderate coughing decreased coughing to mild by 6 months, both of which were euthanized before 12 months. In the remaining 2, coughing decreased to mild in 1 by 12 months and persisted until last follow‐up over 60 months post‐implantation and decreased to mild in the other by 24 months before stopping by 36 months post‐implantation. Of the 3 dogs with mild coughing at 3 months, 1 was euthanized before 12 months with no change, 1 resolved by 12 months, and 1 persisted until last follow‐up over 48 months later.

Abrupt cessation of mild coughing was noted in 3 dogs. Further investigation determined that 2 had broken leads, and these were replaced (Figure 1B,D). One dog (B) also had the generator replaced at the same time. The remaining dog had normal impedance after testing and the VNS was considered to be functioning normally.

Mean monthly seizure days for each dog over time are shown in Table 1. When compared with the 6‐month baseline period, the 50% responder rates were 2/12 during 0 to 6 months (7 worsened, 5 improved), 1/8 during 7 to 12 months (6 worsened, 3 improved), 2/7 during 13 to 24 months (3 worsened, 4 improved), 4/7 during 25 to 36 months (7/7 improved), 4/5 during 37 to 48 months (5/5 improved), 3/4 during 49 to 60 months (4/4 improved), and 2/2 during 60 to 72 months post‐implantation. At their last follow‐up (median, 907 days; range, 139‐2101), 7/12 (58%; 95% confidence interval [CI], 32‐81%) dogs were 50% responders. For dogs surviving >12 months, this proportion was 7/7.

**TABLE 1:** Mean pre‐ and post‐implantation monthly seizure days for dogs included in the study.

Time period	A	B	C	D	E	F	G	H	I	J	K	L
−6 to 12 months	1.8	0.5	1.3	—	1.3	2.6	1.1	1.1	3.4	—	2.4	1.6
−0 to 6 months	2.9	1.1	1.3	3.2	1.3	2.0	1.8	3.9	2.6	4.1	4.4	3.1
0 to 6 months	(3.4)	(2.0)	(3.8)	4.3	**0.5**	(2.3)	**0.7**	(5.1)	(4.7)	2.7	3.1	2.8
7 to 12 months	1.6	1.0	(1.6)	6.8	1.6	(4.0)	**0.8**	(6.5)	—	—	—	—
13 to 24 months	2.8	(1.2)	(1.5)	3.8	**0.5**	(3.0)	**0.4**	—	—	—	—	—
25 to 36 months	**1.6**	0.7	0.8	1.9	**0.0**	**1.1**	**0.7**	—	—	—	—	—
37 to 48 months	**1.5**	0.7	**0.5**	**1.5**	—	**0.5**	—	—	—	—	—	—
49 to 60 months	**0.9**	0.7	—	**1.6**	—	—	—	—	—	—	—	—
61 to 72 months	**0.0**	**0.0**	—	—	—	—	—	—	—	—	—	—

*Note*: Dogs A to G correspond to graphs A to G in Figure 1. Dogs H to L were euthanized before 12 months post‐implantation. A dash indicates a period where data were unavailable for >50% of the period because of euthanasia, last follow‐up or incomplete seizure records. Mean values in parentheses are more than the 6‐month pre‐implantation baseline and mean values in bold type are >50% smaller.

**FIGURE 2:**
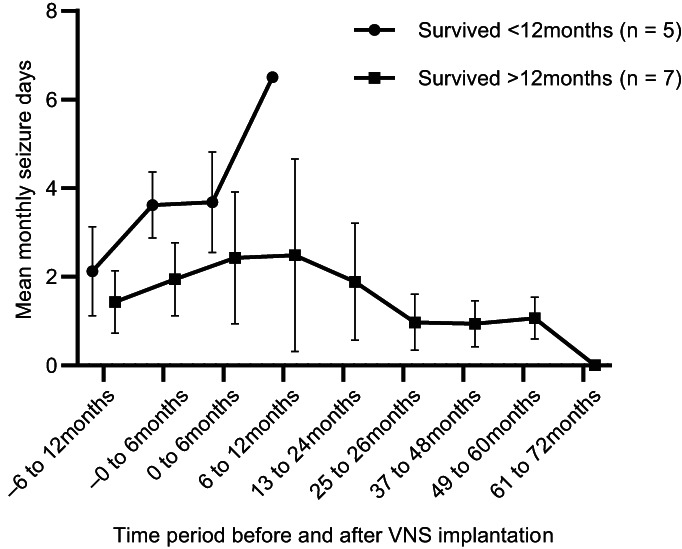
Mean monthly seizure days for dogs euthanized before 12 months post‐implantation and those surviving >12 months. Symbols represent total group means and error bars represent the SD.

Three of the 5 dogs euthanized before 12 months had improved seizure frequency in the first 6 months post‐implantation compared with 2/7 that were not euthanized. In all dogs, mean seizure day frequency increased before and for the 12 months after implantation before appearing to decrease, but this was not analyzed statistically (Figure 2). For the dogs that were euthanized in the first year post‐implantation, median 6‐month pre‐implantation seizure frequency was 3.9 seizures per month (range, 2.6‐4.4) and 1.8 (range, 1.1‐3.2) in those that were not euthanized, whereas median frequency in the 6 months post implantation was 3.1 (range, 2.7‐5.1) seizures per month for those euthanized and 2.3 (range 0.5‐4.3) for those that were not euthanized.

**FIGURE 3:**
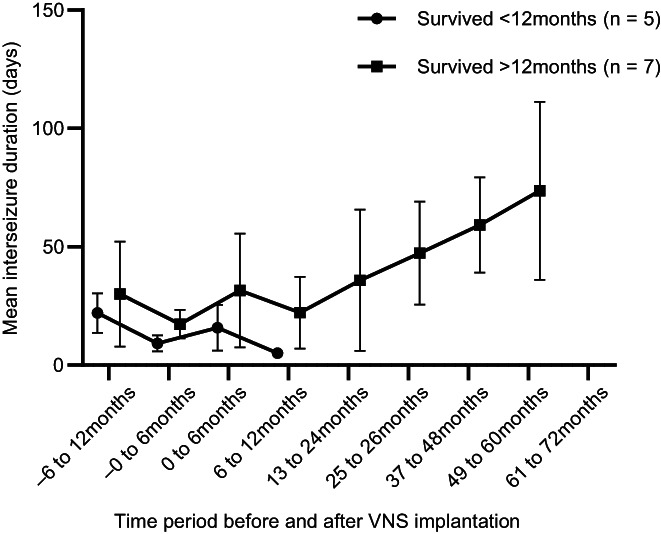
Mean inter‐seizure interval in days for dogs euthanized before 12 months post‐implantation and those surviving >12 months. Symbols represent total group means and error bars represent the SD.

Mean inter‐seizure intervals are shown in Figure 3. When compared with the 6‐month baseline period, the proportion of dogs with a >3 times increase in average inter‐ictal interval was 1/12 at 0 to 6 months (2 decreased and 10 increased), 0/8 at 7 to 12 months (4 decreased and 8 increased), 2/7 at 1 to 2 years (2 decreased and 5 increased), 4/7 at 2 to 3 years (all increased), 3/4 at 3 to 4 years (all increased), 3/3 at 4 to 5 years, and 2/2 at 5 to 6 years (Table 2).

**TABLE 2:** Mean pre‐ and post‐implantation seizure‐free intervals (days) for dogs included in the study.

Time period	A	B	C	D	E	F	G	H	I	J	K	L
−6 to 12 months	16	73	29	—	27	11	24	33	16	—	15	24
−0 to 6 months	15	27	22	8	15	18	16	8	10	4	13	11
0 to 6 months	16	35	(16)	10	77	19	48	7	11	**16**	32	13
7 to 12 months	15	31	36	(5)	(9)	(14)	45	5	—	—	—	—
13 to 24 months	18	30	(17)	15	**72**	(14)	**85**	—	—	—	—	—
25 to 36 months	**54**	67	**76**	**25**	**277** ^a^	25	37	—	—	—	—	—
37 to 48 months	**48**	71	**86**	**34**	—	**57**	—	—	—	—	—	—
49 to 60 months	**84**	**105**	—	**32**	—	—	—	—	—	—	—	—
61 to 72 months	**342** ^a^	**443** ^a^	—	—	—	—	—	—	—	—	—	—

*Note*: Dogs A to G correspond to graphs A to G in Figure 1. Dogs H to L were euthanized before 12 months post‐implantation. A dash indicates a period where data were unavailable for >50% of the period because of euthanasia, last follow‐up, or incomplete seizure records. Intervals in parentheses are shorter than the 6‐month pre‐implantation baseline and intervals in bold type are >3 times longer.

^a^ Indicates seizure freedom for 3 months or > 3 times the longest previous interictal period until death, euthanasia, or end of follow‐up.

Three dogs experienced >3 months or 3 times their previous longest inter‐ictal period before euthanasia for reasons unrelated to their epilepsy or by the end of the study period, which was consistent with a previous definition of seizure freedom.^2^ One was euthanized for progressive paraparesis 5 years and 5 months post‐implantation after 342 days without a seizure; 1 was last followed up 5 years and 10 months post‐implantation after 443 days without a seizure, and 1 was euthanized 2 years and 2 months post‐implantation because of arthritis after 277 days without a seizure.

## 4 DISCUSSION

All dogs continued to seizure post‐implantation and current ramping, showing that VNS is not a rapid, complete cure for dogs with epilepsy. However, our data suggest that substantial improvement can occur in some dogs >12 months after VNS implantation.

When compared with the 6 months before implantation, all 7 dogs surviving >12 months eventually showed 12‐month periods of >50% decrease in their seizure‐day frequency, 6/7 showed 12‐month periods of a >3 times increase in their inter‐seizure interval at the time of euthanasia or last follow‐up, and 3/7 achieved seizure freedom.

Vagus nerve stimulation was well tolerated. In the 11 dogs that reached 1.5 mA, 4 did not cough, 3 had a mild cough, and 4 had a moderate cough. Coughing decreased with time in most dogs and was considered tolerable even when persistent. Lead replacement was required in 2/12 dogs, which is similar to previous findings in dogs where 2/10 had lead damage 22 days and 5 months after implantation,^10^ but lower than reported rates in humans of 30% (95% CI, 23%‐38%), although this difference may reflect the longer follow‐up of 4.9 years in the human cohort.^14^ One of 12 of our dogs had generator replacement because of malfunction, which we believed was secondary to lead damage. We were unable to find similar reports in dogs or an estimated rate in humans.

One limitation of our study was its design. Before and after studies such as ours (where an outcome measure is assessed before and after an intervention) are commonly used to investigate treatments for epilepsy in dogs.^3^ They are thought to be limited because they cannot infer causality between an effect seen and an outcome measured and tend to over‐estimate treatment effects for 2 main reasons: temporal changes and regression to the mean.^15^

Temporal changes refer to the natural variation of disease severity, meaning that if the natural course of epilepsy in dogs is to decrease in severity and eventually stop in some dogs over several years, then this effect could be mistakenly attributed to an intervention. Few studies report seizure frequency in dogs over >18 months,^3^ which highlights the paucity of research in this area despite the a priori definition of epilepsy as an enduring tendency to have seizures.

Regression to the mean refers to the phenomenon that severity after an intervention is likely to decrease because increased severity prompted the intervention, meaning the pre‐intervention increase could have been a chance fluctuation and therefore not a true measurement of actual disease severity. This scenario has been suggested to be a common cause of decreased seizures in before and after trials in dogs.^16^

We attempted to minimize the regression effect in 3 ways. First, we used a baseline period of 6 months instead of a more typical 3‐month period,^3^ which we hoped would provide a more accurate reflection of monthly seizure frequency. Second, we used a long follow‐up period to minimize fluctuations over time. Finally, we used a modified rule of 3 when assessing the increase in inter‐seizure interval because it has been suggested to account for chance variation in seizure frequency.^13^

The most effective way to overcome these limitations is to use a randomized, controlled trial design (RCT). A RCT in dogs failed to show a significant reduction in seizure frequency compared with control dogs over 13 weeks after implantation.^7^ Three published RCTs in humans did show significant decreases in seizure frequency 3‐months post‐implantation, but not a significant increase in 50% responders,^17^ This outcome may support the argument that the effectiveness of VNS seen in observational before and after studies is associated with a flawed study design, but also may be that the follow‐up periods are too short given that seizure decreases post‐implantation appear to occur over several months to years in epileptic dogs and humans.^17^, ^18^

The reason for this delayed decrease in seizures is unclear. One hypothesis is that the formation of new synapses between neurons in response to altered neurotransmitter release can take several months.^5^ Another is that the VNS might be able to modulate the inflammatory response in the brain, which is thought to play a key role in epilepsy.^19^

Without a RCT and an appropriate control or placebo group, it might be difficult to determine if the initial post‐implantation increase in seizures and then subsequent decrease is caused by the natural course of the disease and independent of implantation or caused by the VNS itself. A RCT may be hard to perform, but our findings indicate a clear need for more research into the natural history of epilepsy in dogs.

Another limitation was the small number of dogs with appropriate follow‐up either because of failure to keep a seizure diary or euthanasia before 12 months post‐implantation. The reasons why seizure diaries were not maintained were not established and we were not able to find comparative rates in other veterinary studies. This area would be valuable for further research, and we recommend in future trials that a more consistent follow‐up schedule and a standardized recording method (eg, a phone app) might improve record keeping.

We did not establish a clear reason why some dogs were euthanized before 12 months and some were not. Monthly seizure frequency decreased at 3 months post‐implantation in 3/5 dogs that were euthanized before 12 months and in 2/7 in those who were not euthanized, suggesting failure to improve was not the only risk factor, or that early improvement was a strong protective factor. We recruited dogs with a high rate of monthly seizures, and our data suggest that this rate might have been highest in the dogs that were euthanized, suggesting severity of epilepsy might be a strong risk factor for early euthanasia. Further research is required in this area to identify risk factors that indicate which dogs have a high risk of euthanasia before any effect of VNS can be seen.

Another limitation was that we allowed medication changes after implantation. This design feature could confound any effect of VNS if a novel antiseizure drug was used. Only 3 dogs had novel medications started and all eventually showed a 50% decrease in seizures. In 2 (Figure 1A,D) this decrease occurred several months to years after starting zonisamde and imepitoin, respectively, making a relationship unlikely, but in the other dog (Figure 1G) the 50% decrease occurred in the same month as VNS implantation and starting pregabalin, making the relationship very plausible. We did not believe it was ethical to deny medication changes without evidence of VNS effectiveness in dogs, and there is some evidence that any impact in trials conducted in humans is minimal,^20^ but we would recommend that medication changes are controlled in future trials.

We might have introduced inaccuracy by including data for dogs where up to 50% of a seizure diary was missing for a 6‐ or 12‐month period. We did so because we did not want to exclude dogs if some data were missing and anticipated that keeping diaries over several years might be difficult. This concern was corroborated by the fact that 4/16 owners of eligible dogs did not maintain a seizure diary post‐implantation. Only 1 dog (D) we included had missing data, and it occurred during 25 to 36 months post‐implantation when its seizure days were not reported as >50% decreased or the interval was >3 times longer than baseline, suggesting that including data for this period did not artificially increase the responder rate, but may have decreased it.

## 5 CONCLUSION

Implantable VNS were tolerable and >50% decrease in monthly seizure frequency and >3 times increase in inter‐seizure interval was seen in all dogs that survived >12 months post‐implantation. These changes took several months or years in some cases, and the rates might have been lower if the outcome for the dogs euthanized before 12 months could have been included. Further research is indicated to see if these results are part of the natural history of medication‐resistant epilepsy in dogs, or the effect of VNS, but the design of these trials may be problematic. Further work also is required to identify what protective or risk factors influenced owners' decisions about euthanasia to aid future recommendations about a treatment that has an effect over several months to years in cases where it is beneficial.

## CONFLICT OF INTEREST DECLARATION

Thomas R. Harcourt‐Brown and Michael Carter attended a meal paid for by Livanova to discuss implanting VNS in dogs and received technical advice about the use of the devices from the firm. Livanova also provided some of the equipment for free or a discounted price. There was no involvement from Livanova in the collection of data or the writing of the manuscript. Thomas R. Harcourt‐Brown has been approached by a company developing a canine specific vagus nerve stimulator who has asked him to be part of their advisory board. That company had no involvement in the data collection or preparation of the manuscript.

## OFF‐LABEL ANTIMICROBIAL DECLARATION

Authors declare no off‐label use of antimicrobials.

## INSTITUTIONAL ANIMAL CARE AND USE COMMITTEE (IACUC) OR OTHER APPROVAL DECLARATION

Authors declare no IACUC or other approval was needed. approval granted by the University of Bristol animal welfare and ethics review body (AWERB) under the unique veterinary investigation number VIN/20/035.

## HUMAN ETHICS APPROVAL DECLARATION

Authors declare human ethics approval was not needed for this study.

## Supporting information


**Data S1.** Supporting Information.
