# Supplementary material for: Long‐term outcome of epileptic dogs treated with implantable vagus nerve stimulators
Source: J Vet Intern Med. 2023 Oct 20;37(6):2102–8. doi: 10.1111/jvim.16908 (PMC10658546; doi:10.1111/jvim.16908)
Supplement: Supplementary file 1 — Data S1. Supporting Information. [file JVIM-37-2102-s001.pdf]

Medication changes in dogs

	Phenobarbital	Potassium bromide	Levetiracetam	Zonisamide	Gabapentin	Pregabalin	Imepitoin	CBD oil	Changed
A	y	Y - Stopped @41 days because of bromism	y	Started at 41 days	Started @41d				Yes
B	y	y	Y - Pulse therapy until day 755 post implantation				Pulse from day 755 post implantation	Y	Yes
C	y	y	y						No
D	y	y	Y - stopped 450 days post implant because ineffective				Started at 121 days post implant		Yes
E	Y	Y							No
F	y		y	y			y		No
G	y	No - developed bromism		y	Started 83 days post implantation				Yes
H	y	y	y						No
I	y	y	y						No
J	Y	Y	Y						No
K	y	y	y				y		No
L	Y	No - too sedated	Y		Y				No

Medications in dogs. Y = on an appropriate dose or therapeutic serum concentration at the start of the study. No = Did not tolerate medication and discontinued prior to start of study. Text in boxes indicates when a drug was started or discontinued during the study period.
